# A Generalized Software Framework for Consolidation of Radiation Therapy Planning and Delivery Data From Diverse Data Sources

**DOI:** 10.1016/j.adro.2026.102134

**Published:** 2026-07-11

**Authors:** Yasin Abdulkadir, Justin Hink, Peter Boyle, Dishane Luximon, Justin Pijanowski, Timothy Ritter, Bruce Curran, Min Leu, Nicholas Nickols, Steve P. Lee, Maria Kelly, Byron Wilson, Zachary Barton, Jatinder R. Palta, Rishabh Kapoor, Reid F. Thompson, Daniel A. Low, James M. Lamb, Jack Neylon

**Affiliations:** a Department of Radiation Oncology, University of California, Los Angeles, California; b Virginia Commonwealth University Health System, Department of Radiation Oncology, Richmond, VA; c VA Richmond Healthcare System, Department of Radiation Oncology, Richmond, VA; d VA Greater Los Angeles Healthcare System, Department of Radiation Oncology, Los Angeles, CA; e VA Long Beach Healthcare System, Department of Radiation Oncology, Long Beach, CA; f Department of Veterans Affairs National Radiation Oncology Program, Richmond, VA; g Oregon Health and Science University, Department of Radiation Medicine, Portland, OR; h VA Portland Healthcare System, Department of Radiation Oncology, Portland, OR

## Abstract

**Purpose:**

Large-scale radiation therapy data sets drive predictive modeling, automated segmentation and planning, and personalized treatment, yet remain fragmented because treatment planning (TPS) and record-and-verify (R&V) systems differ, Digital Imaging and Communications in Medicine (DICOM) implementations are inconsistent, and automated linkage tools are lacking. We developed a generalizable framework that automatically reconstructs complete planning and delivery data sets across diverse clinical environments with minimal manual effort.

**Methods and Materials:**

We designed and implemented a software framework capable of automating the collection and integration of radiation therapy data from multiple institutions and TPS/R&V combinations. The system begins with Radiation Therapy Treatment Records and recursively traces unidirectional DICOM references to retrieve linked radiation therapy treatment plans, doses, structure sets, planning images, image registrations, and associated diagnostic images. Core components of the framework include automated DICOM queries, secure data transfer, integrity verification, linkage mapping, and detailed logging. To support diverse environments, we developed custom modules for non-DICOM-compliant systems, file format conversions, and robust error handling.

**Results:**

The framework was deployed across 4 institutions using 6 different combinations of TPS and R&V systems. In a focused 2-clinic implementation spanning 11 years of retrospective data, the system successfully processed and integrated data from 6164 patients and 13,871 radiation therapy plans. The pipeline achieved a 99.76% success rate in identifying and linking complete treatment data sets, with an average processing time of 18 minutes per patient, demonstrating its efficiency and scalability in real-world conditions.

**Conclusions:**

This automated framework provides a scalable and reliable solution for large-scale aggregation of radiation therapy data. It is compatible with heterogeneous clinical systems, including those lacking DICOM Query/Retrieve support, and overcomes key technical barriers to data integration. By enabling the creation of comprehensive, high-quality data sets, the framework supports advanced research and contributes to the improvement of clinical care in radiation oncology.

## Introduction

Very large databases of radiation therapy planning and delivery data have immense potential to advance radiation oncology research and clinical practice. Such “radiation therapy big data” can facilitate the development and validation of predictive models, enhancing outcomes and treatment efficiency,^1^ and can reveal patterns and correlations that smaller data sets cannot, thereby improving the precision and personalization of radiation therapy treatments.^2^ Additionally, radiation therapy big data may enable artificial intelligence-based automated segmentation and radiation therapy planning, allowing improved quality, safety, and efficiency of treatments, and potentially leveraging the expertise of tertiary providers to under-resourced clinics that treat underserved patients.

Several initiatives have made significant strides in the retrospective collection of radiation therapy data. The Cancer Imaging Archive (TCIA),^3^ for instance, hosts a vast array of imaging data sets, including those in Digital Imaging and Communications in Medicine for Radiation Therapy (DICOM-RT) format, demonstrating the feasibility and utility of aggregating radiation therapy data from diverse sources. TCIA relies on contributing sites to curate and submit data, ensuring its consistency and usability.^4^ However, as of June 16, 2024, TCIA contains only ∼12,000 radiation therapy patient data sets with labeled anatomy (ie, radiation therapy structure set [RTSTRUCT]) and ∼2000 patient data sets with radiation treatment plan dose distributions (RTDOSE). Another notable initiative is the National Radiation Oncology Registry, which was established to collect and analyze radiation treatment data on a large scale, promoting evidence-based practices.^5^ The University of Michigan’s Radiation Oncology Analytics Resource exemplifies a successful effort in aggregating key multidisciplinary data elements, supporting clinical and research applications with data for over 17,000 patients treated since 2002.^4^

Despite the progress made thus far, we and others believe that even larger databases would provide increased benefit to radiation therapy research. For example, the creation of ImageNet,^6^ containing 14 million manually labeled images, was instrumental in the development of image classification algorithms that have seen widespread public usage. However, the technical challenges in creating radiation therapy big data enterprises are considerable. Assembling such data requires merging multiple disparate clinical data sources, necessitating effective strategies for standardizing and integrating these data into a cohesive database. Ensuring data interoperability requires robust frameworks for data standardization and normalization. Furthermore, the sheer volume of data necessitates advanced storage solutions and efficient data management systems capable of handling large-scale data sets.^7^ Maintaining data quality and completeness is crucial, as inconsistencies and gaps in data can lead to erroneous analysis and conclusions. Manual data quality validation and correction mechanisms are impractical, so automated processes are needed. Compliance with regulations such as Health Insurance Portability and Accountability Act of 1996 (HIPAA) requires stringent security protocols to protect patient information.^8^

Here, we describe the architecture and report the initial performance of a software framework designed for the automated collection of multi-institutional planning data stored in diverse treatment planning systems (TPS) and record-and-verify (R&V) systems. Our primary objective was to gather data corresponding to treated radiation therapy plans only, which had important implications for the software design, described in the Methods section. The framework was built to operate efficiently, ensuring timely data collection and processing. It includes mechanisms to handle errors and interruptions gracefully, maintaining continuous operation. In addition, it verifies the delivery of radiation therapy treatment plans (RTPLAN) to exclude incomplete or irrelevant data, such as unused trial plans; interfaces with multiple planning systems, including those without DICOM interfaces; and manages diverse data formats and sources to ensure comprehensive data integration. This robust and versatile framework aims to streamline the aggregation of radiation therapy data, thus facilitating large-scale analyses and fostering advancements in treatment quality and safety.

## Methods and Materials

### Data collection and framework structure

Each DICOM object has a unique identifier (UID). References to these UIDs create linkages between objects. However, these references are rarely reciprocal, limiting the recreation of the DICOM data structure for a patient to a unidirectional path. The DICOM structure followed in the code involves several key components:

• Radiation Therapy Treatment Record (RTRECORD): the starting point for data collection, containing references to the RTPLAN.
• RTPLAN: linked from RTRECORD, it contains treatment planning information.
• RTDOSE: connected to RTPLAN, it contains dose distribution data.
• RTSTRUCT: linked from RTPLAN, it contains structure set data (contours).
• Planning computed tomography (CT)/magnetic resonance (MR): connected to RTSTRUCT, it contains the planning imaging data.
• Registration (REG): registration data that links planning images to other modalities like CT, MR, or positron emission tomography (PET).
• Registered CT/MR/PET: imaging data registered to the planning images.

The logical processing algorithm for this framework is referred to as the “CORE,” which centrally manages tasks like data queries, transfers, verification, and logging. The linear steps taken to map the DICOM linkages and gather the objects from a DICOM-compliant interface are as follows, starting at the point of a successful query of an RTRECORD from an R&V system:

1. Move the RTPLAN referenced by returned RTRECORDs to the CORE, by UID.
2. Move the RTSTRUCT referenced by the RTPLAN to the CORE, by UID.
3. Query/retrieve (Q/R) the RTDOSE that references the RTPLAN.
4. Q/R all RTRECORDs referencing the RTPLAN.
5. Q/R the CT (Simulation) SIM slice that is referenced by the first slice of the first nonempty structure in the RTSTRUCT.
6. Q/R the CT SIM series to which the slice identified in step 5 belongs.
7. Q/R the REG that references the CT SIM slice.
8. Q/R for all CT/MR/PET series that share the same Frame of Reference UID with the CT SIM.
9. Q/R the diagnostic images referenced by the REGs that reference the CT SIM.
10. Q/R all images that share a Frame of Reference UID with images identified in step 9.

**Figure 1:**
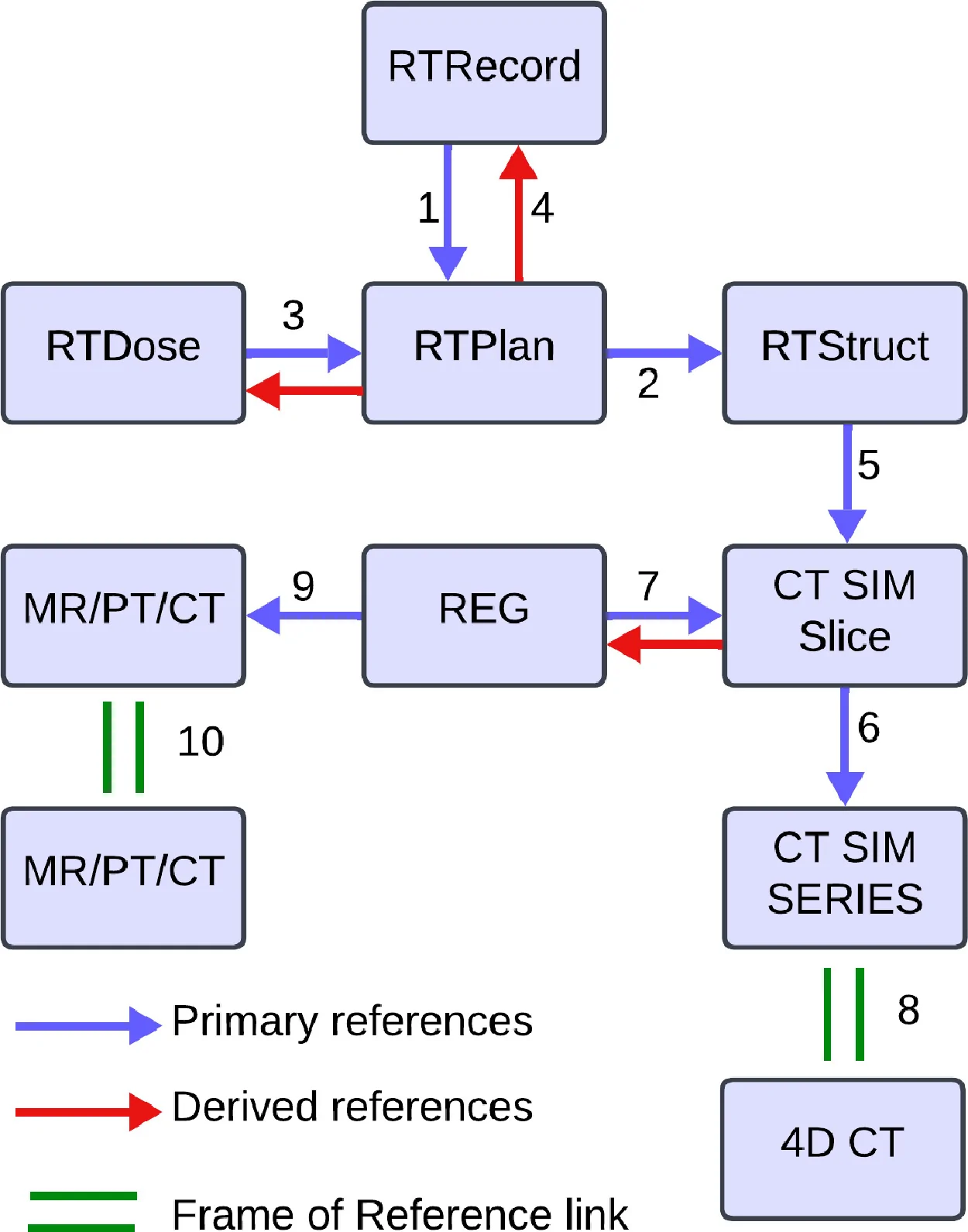
DICOM-RT connectivity used and defined by our software framework. Blue lines indicate native references stored within DICOM-RT files. Red lines indicate references derived by our code (ie, by reverse lookup). Numerals indicate the order of steps within the process to reconstruct the entire object chain (as described in the Data collection and framework structure section). *Abbreviations*: CT = computed tomography; DICOM-RT = DICOM for Radiation Therapy; MR = magnetic resonance; PT, Positron Emmision Tomography; RTDOSE = radiation treatment plan dose; RTPLAN = radiation therapy treatment plan; RTRECORD = radiation therapy treatment record; RTSTRUCT = radiation therapy structure set; REG = registration; SIM = Simulation.

Figure 1 presents a graphic depiction of DICOM objects representing radiation therapy planning and delivery, the linkages between them, and the steps outlined above to map the linkages between the objects. Once each object was identified, it was queued for DICOM transfer to a Picture Archiving and Communication System (PACS) that we set up. Before moving an object, it was verified through Q/R that it was not already in the PACS. Because our primary objective was to gather data corresponding only to treated radiation therapy plans, RTRECORD was made the starting point for assembling a patient data set. By initiating the data collection with the RTRECORD, we ensured that the data set was anchored in an administered treatment. To streamline this process, we developed methods to inspect DICOM reference connections, which facilitated the identification and linkage of relevant data objects. This approach minimized unnecessary data transfers by ensuring that only essential and directly connected data were collected. However, there is added practical complexity because intuitive concepts of belongingness are not always reflected in DICOM references. For example, in most TPS systems, the RTPLAN object does not contain a pointer to an RTDOSE object; rather, the RTDOSE object contains a pointer to the RTPLAN object (its SOPInstanceUID). Therefore, to find the RTDOSE that corresponds to RTPLAN, it was necessary to search through all the RTDOSE objects with the same StudyInstanceUID (the parent object that both RTPLAN and RTDOSE belong to) as the RTPLAN and determine which had a pointer to the RTPLAN. These DICOM reference relationships are represented as red arrows in Figure 1. Finally, in clinical treatment planning software, when registering a PET/CT to a simulation CT, an explicit REG object is created for the CT-CT registration, but the PET-CT registration is based on the shared frame of reference, meaning that the PET and CT images acquired during the PET/CT scan inherently share the same coordinate system. This shared frame of reference allows the PET and CT images to be automatically aligned without the need for additional registration processes between them. Thus, when attempting to gather all diagnostic images registered to the simulation CT, it is necessary to obtain the diagnostic images that are referenced by REG objects, as well as all diagnostic images that share a FrameOfReferenceUID with explicitly referenced images.

### Data sources

**Figure 2:**
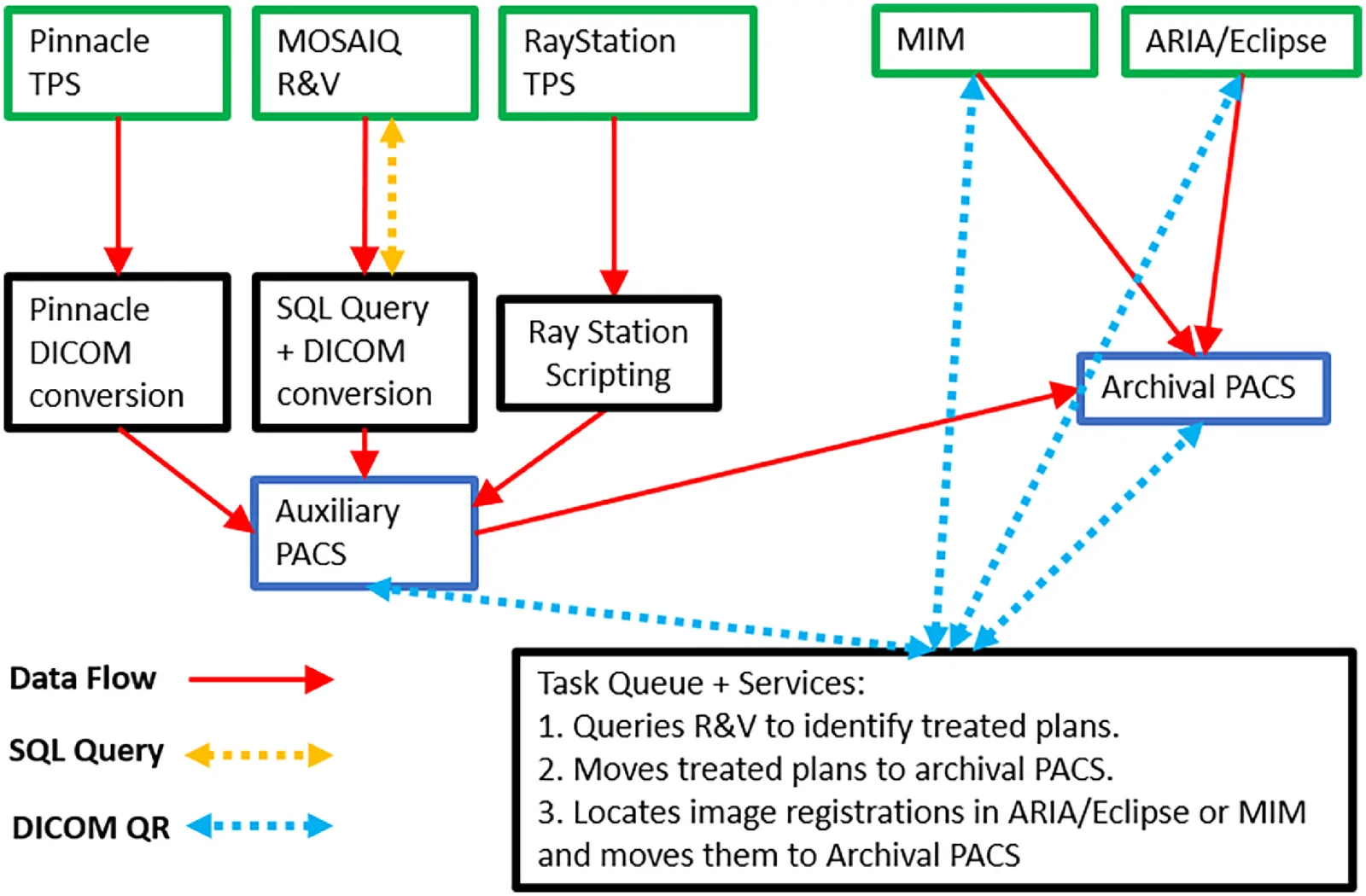
Diagram of data sources, code modules, and the archival data repository. Data sources are boxed in green, program modules are boxed in black, and PACS are boxed in blue. *Abbreviations*: ARIA (Siemens Healthineers) is an Oncology Information System; DICOM = Digital Imaging and Communications in Medicine; MIM (GE Healthcare) is a PACS; PACS = Picture Archiving and Communication System; QR = Query and Retrieve; R&V = record-and-verify system; SQL; TPS = treatment planning system.

The system was designed to gather treatment planning and delivery data from multiple institutions employing various TPS and R&V systems. Systems from which we collected data included ARIA and Eclipse (Siemens Healthineers), MOSAIQ (Elekta, Inc), RayStation (Raysearch Laboratories), MIM (GE Healthcare), and Pinnacle (Phillips). Where possible, vendor-supported DICOM interfaces were used (ARIA/Eclipse, MIM, RayStation). Explicit conversion to DICOM was required for data from Pinnacle and MOSAIQ installations. Figure 2 shows a diagram of data sources and how they are interfaced to program modules and mapped to the archival data repository.

### Software framework architecture

The software framework developed for this study incorporates several critical components designed to ensure efficient and robust data collection from multiple sources.

#### Job-queuing system

**Figure 3:**
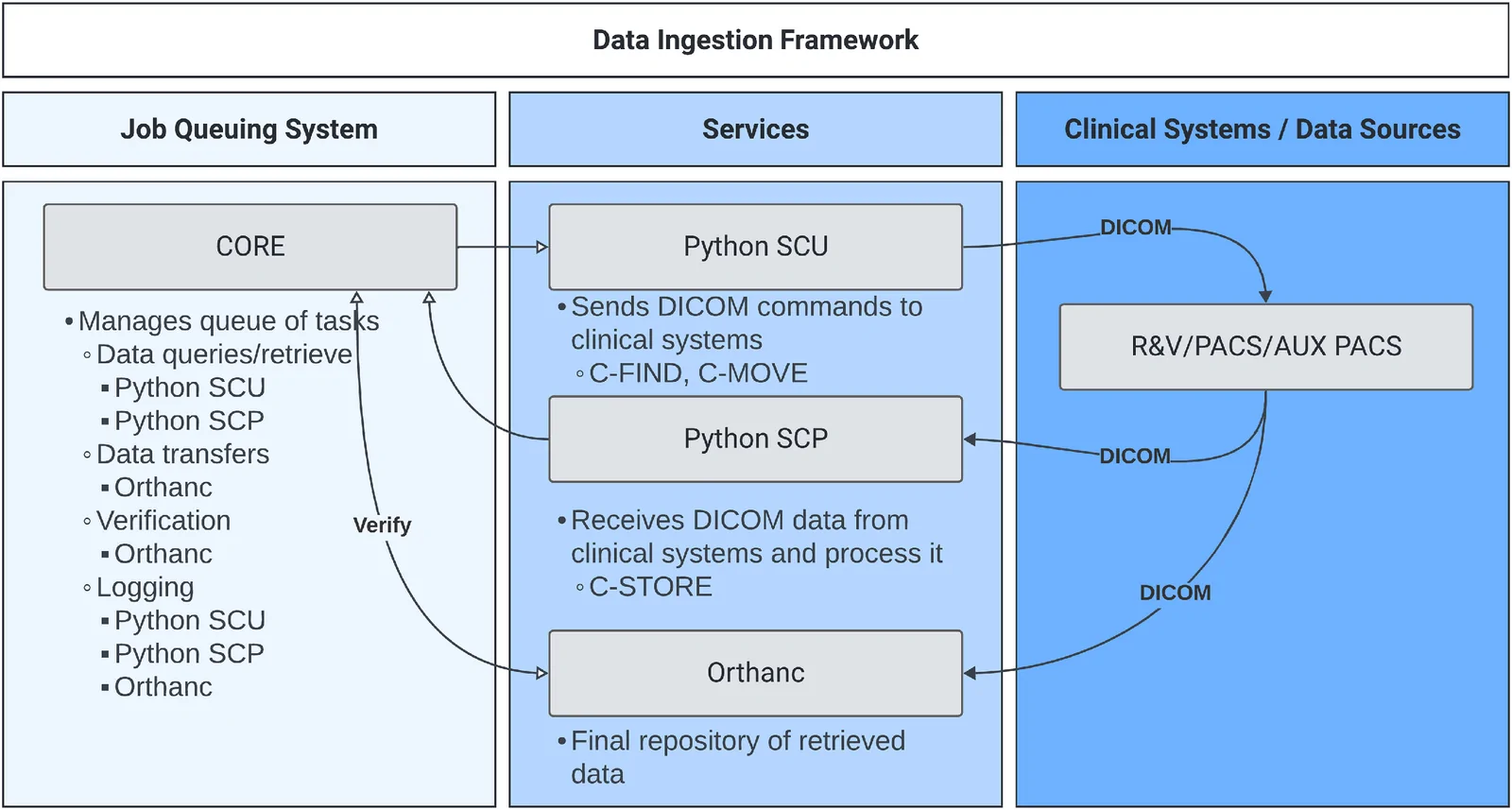
Data ingestion workflow. The “CORE” centrally manages jobs like data queries, transfers, verification, and logging. The Python SCU communicates with clinical systems using DICOM commands (C-FIND, C-MOVE), whereas the Python SCP handles incoming DICOM data via C-STORE and generates jobs for the queue. “Orthanc” serves as the “archival PACS” or final repository, ensuring data integrity and accessibility. *Abbreviations*: AUX = Auxiliary; C-FIND = Composite Find; C-MOVE = Composite Move; C-STORE = Composite Store; DICOM = Digital Imaging and Communications in Medicine; PACS = Picture Archiving and Communication System; R&V = record-and-verify system; SCP = Service Class Provider; SCU = Service Class User.

Central to the architecture, this system manages the collection process. Each job represents a DICOM object type and a corresponding set of actions. A Job Queue stores and processes jobs, ensuring efficient data handling and minimizing data transfers by inspecting DICOM reference connections. Figure 3 illustrates the job-queuing system, its functionality, and its relationship to DICOM sources.

#### Non-DICOM queryable data transfers

The MOSAIQ R&V, Pinnacle TPS, and RayStation TPS lack DICOM queryable transfer interfaces. In a typical Pinnacle installation, most saved data are stored as proprietary formats bundled as “tar” files. RayStation allows scripted export of DICOM-formatted plans but does not support DICOM query-retrieve. Our approach was to write dedicated code and processes to send these data (converting to DICOM if needed) to what we termed an auxiliary PACS, which then was interfaced to our main job-queuing system. This was done to provide a single framework that connected treatment plan objects with the information about which plans were treated, keeping in mind that the overall goal was to store only treated plans and retain the information on the number of fractions that each plan was treated.

#### Mosaiq DICOM conversion module

MOSAIQ provides treatment data (analogous to what is found in an RTRECORD) via (Structured Query Language) SQL interface. DICOM RTRECORDs are not provided without the DataDirector purchasable option, which many clinics have not purchased. Thus, a software module was written to extract treatment information via SQL and write RTRECORDs.

#### Pinnacle tar file conversion

We converted Pinnacle Tape Archive (TAR) files using modified open-source code from the PyMedPhys project.^9^ Our enhancements allowed processing of multiple patients per TAR file and bulk directory processing. We also ensured the generation of DICOM-compliant UIDs with correct reference relationships. In addition, we modified hard-coded tags to be automatically collected and implemented mechanisms to gracefully handle errors, logging them for manual processing.

#### MIM sessions data

MIM allows the possibility to store radiation therapy structure sets and image registrations in a *Sessions* format. Although in the MIM Patient List User Interface, structure sets and registrations appear to be stored as RTSTRUCT and REG objects, internally they are stored as DICOM RAW objects. Within the RAW objects, private tags hold DICOM header information identical to that found in RTSTRUCT and REG headers. Thus, we developed code to unpack the Sessions and convert the contents to RTSTRUCT and REG objects.

#### Unit and integration testing

Pytest^10^ was used for both unit and integration tests. Extensive tests were conducted on individual modules to ensure each component operates correctly. Integration tests were conducted to verify that the entire system functions as expected when all components are combined.

#### Data storage and management

All data, from whatever source, was required to be ultimately aggregated into a single, cohesive database. The Orthanc open-source PACS^11^^,^^12^ was selected as our archival PACS. Orthanc was chosen for several reasons. First, we felt it was essential that the data were stored in a PACS to facilitate further processing and/or transfer to a larger downstream database because PACS systems are queryable and manage duplicate data storage requests transparently. Secondly, Orthanc has an integrated SQL database that allows for the storage of ancillary information on a per-object basis in a way that is transparent to the end user (eg, we chose to add information on the provenance of each DICOM object), and also supports the REST API which allows our custom software to make calls directly to the underlying Orthanc program (ie, at a more direct level than performing DICOM queries).

#### Error handling and logging

Error handling ensures robust operation despite potential issues during data collection and processing. Logging provides detailed documentation of all activities related to data extraction, transformation, and storage, and allows for efficient recovery from crashes due to network or hardware issues (eg, computer being turned off) that were inevitable over multiple weeks of running. Comprehensive error handling routines are incorporated, capable of identifying, logging, and recovering from errors. These routines include retry mechanisms and alerts for manual intervention when necessary. Logging mechanisms are integrated within the Python Service Class User (SCU), Python Service Class Provider (SCP), and Orthanc components. Logs include detailed information about data transfer activities, errors, and recovery actions. Logging was implemented by writing to an SQLite database; log reading was performed using SQL queries. All software modules logged errors into the same database. This allowed faster and more robust searching of the extensive log files consisting of millions of logged messages.

#### Data validation and manifest generation program

A software module was created to validate collected data by performing error and consistency checking, and to generate human-readable summaries. This program module inspects DICOM reference connections to identify missing data. It provides summaries to facilitate human review and ensure data completeness.

#### Application and testing

The software framework was designed in consideration of the collection of data from radiation therapy clinics throughout the Department of Veteran's Affairs (VA) health system. An initial phase consisted of a survey of the VA radiation therapy data landscape. Data collection was undertaken at 4 clinics and completed at 2 clinics, as described below.

## Results

### Variability of clinical systems

To better understand the scope and complexity of retrospective data extraction within the Veterans Health Administration (VHA) system, we first sought to explore the archival data landscape across VHA clinics. This exploration aimed to identify the challenges associated with aggregating radiation therapy data from diverse clinical systems, which is crucial for developing an effective data collection framework.

In a survey of nearly 40 clinics, only 31% reported a single data repository for all their radiation therapy data (planning and treatment) from the past decade. Multiple R&V systems were reported at 28% of clinics, multiple TPS were reported at 46%, and 44% of clinics had a separate system for performing image fusions outside of their TPS.

**Figure 4:**
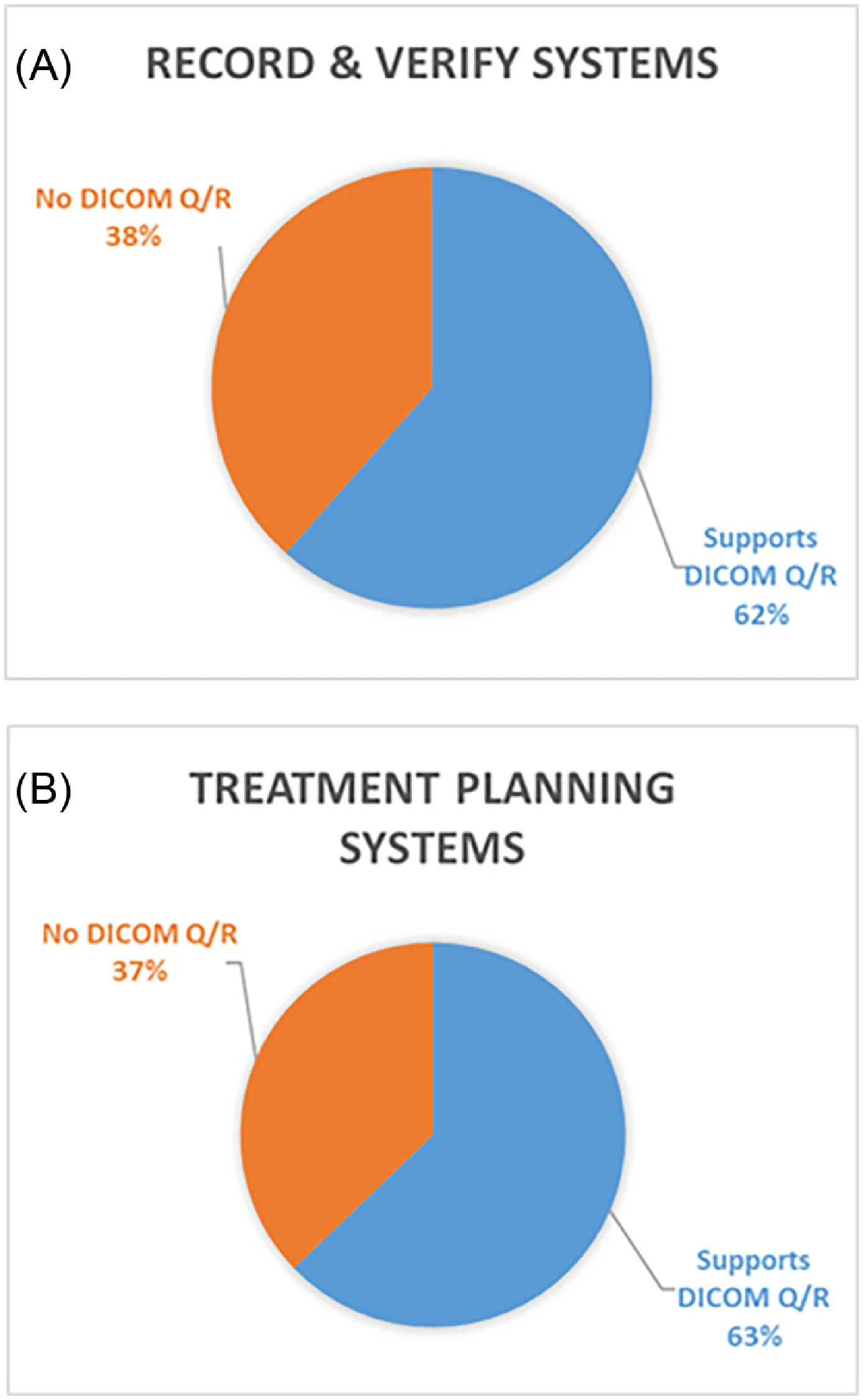
Native support for DICOM Query/Retrieve (Q/R) for clinical systems was self-reported in a survey of nearly 40 radiation therapy clinics. (A) The percentage of clinics that used at least 1 record-and-verify system without DICOM Q/R capability. (B) The percentage of treatment planning systems in use at the clinics without DICOM Q/R capability. *Abbreviation*: DICOM = Digital Imaging and Communication in Medicine.

Figure 4A displays the percentage of clinics with all treatment data in R&V systems that natively support DICOM Q/R, whereas Fig. 4B displays the percentage of self-reported estimates of plans stored in TPS that natively support DICOM Q/R.

### Data collection overview

Data collection was undertaken at 4 deanonymize clinics, which were selected to encompass a range of clinical systems and geographic locations. We successfully completed the collection of radiation therapy planning and treatment data from an 11-year period (2012-2022) from 2 clinics. These 2 clinics employed an ARIA R&V system with treatment plans created in the Eclipse, RayStation, and BrainLAB TPS. Table 1 enumerates the total patients and plans collected during this trial. From both clinics, a total of 6164 patients and 13,904 plans were referenced by treatment records. The framework successfully retrieved 13,871 plans, for a success rate of 99.76%. The small number of plans that were not retrieved was investigated. In many of these cases, the RTRECORD referenced a nonpatient plan or a plan from several years before the designated search window. It took an average of about 18 minutes per patient to process and transfer the data.

**Table 1:** Summary of data collection volumes from the preliminary testing of the proposed data ingestion framework at 2 clinics that primarily used clinical systems that natively support DICOM Query/Retrieve

	Clinic 1	Clinic 2	Total
Unique patients (n)	2629	3535	6164
Unique plans referenced	4784	9120	13,904
Plans collected	4782	9089	13,871
Success rate (%)	99.96	99.66	99.76

*Abbreviation*: DICOM = Digital Imaging and Communications in Medicine.

### Summary of collected data

**Figure 5:**
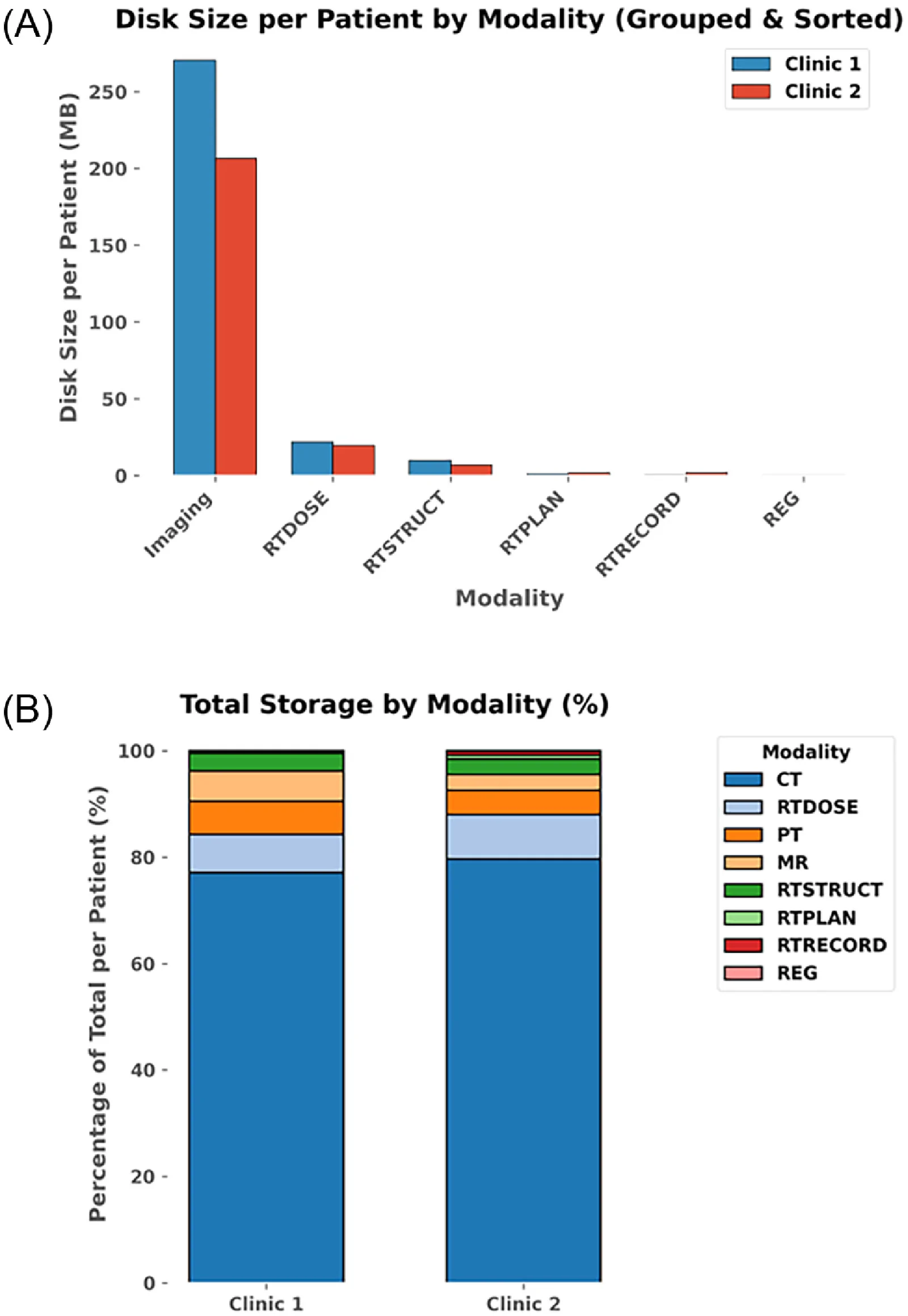
(A) Comparison of average disk usage per patient (in MB) by modality for clinic 1 and clinic 2, showing absolute storage requirements across modalities. Imaging combines the CT, MR, and PT. (B) Total storage contribution of each modality as a percentage of total storage. *Abbreviations*: CT = computed tomography; MR = magnetic resonance; PT, also PET (Positron Emmision Tomography); RTDOSE = radiation treatment plan dose; RTPLAN = radiation therapy treatment plan; RTRECORD = radiation therapy treatment record; RTSTRUCT = radiation therapy structure set; REG = registration.

We collected detailed data on imaging modalities and treatment plans for each clinic. This includes the total number of patients and counts of CT, MR, and PET scans, with a breakdown of CTs referenced by RTSTRUCT, as well as various types of registrations (REG) between imaging modalities, such as CT-CT, CT-MR, and CT-PET. In clinic 1 (Fig. 5), on average, CT volumes comprise 234 MB per patient—about 77% of the total 304 MB storage footprint—whereas RTDOSE contributes 22 MB (∼7%). In clinic 2 (Fig. 5), CT still dominates at 189 MB per patient (∼80% of 237 MB total), with RTDOSE at 20 MB (∼8%). The total memory (clinic 1 = 869 GB, clinic 2 = 817 GB) was grouped by modality and then divided by the total number of patients (see Table 1) to obtain an average memory footprint per patient. These data illustrate that CT storage dominates the per-patient requirements in both clinics, with RTDOSE adding a comparatively minor but nontrivial overhead. The collected data also categorize treatment plans by type—curative, palliative, and prophylactic—alongside counts of absent and invalid plans. Invalid plans are further classified into categories such as verification and machine quality assurance. Although clinic 1 exclusively used the ARIA R&V system with Eclipse TPS, clinic 2 employed additional planning systems. This comprehensive data set provides insights into patient volumes, imaging studies, and treatment plan characteristics for each clinic over the study period.

## Discussion

To fully realize the potential of big data in radiation therapy, it is essential to capture both treatment planning and actual treatment delivery information, as insights from correlations between outcomes and treatments depend on this comprehensive data. However, automating the extraction of such data—while preserving the connections between delivery records (RTRECORDs) and planning information (RTPLAN, RTDOSE, RTSTRUCT, Images)—poses significant challenges. Chief among these challenges is the issue of system variety.

Several prior initiatives have demonstrated the value of large-scale aggregation of radiation therapy data for research, quality assurance, and clinical decision support. National and institutional efforts such as TCIA, National Radiation Oncology Registry, and the University of Michigan’s Radiation Oncology Analytics Resource have shown that centralized radiation therapy data sets can enable population-level analyses, benchmarking, and the development of predictive models. For example, a national radiation therapy data infrastructure has implemented standardized nomenclature and automated pipelines to collect and store structured radiation therapy data in site-level archives that feed a centralized registry; notably, even within this national framework, manual curation remained necessary for a subset of target volumes, underscoring the persistence of heterogeneity challenges.^13^

It is common for clinics to use diverse planning systems and technologies, making it difficult to rely on a single data repository for all relevant radiation therapy information, especially when treatment delivery data are included. This diversity, compounded by the unidirectional references in the DICOM standard, increases the complexity of our CORE logic, which must reconstruct these relationships from disparate sources of delivery and planning data. This process is labor-intensive and can sometimes be counterintuitive. For example, we found that one vendor deviated from the common referencing pattern: while most RT Plans reference an RT Structure Set, in TomoTherapy, the RTDOSE—not the RTPLAN—references the RTSTRUCT.

From this experience, we recommend that the DICOM standard adopt reciprocal referencing to enhance data interconnectedness. However, such an improvement would only be effective if clinical data repositories widely support DICOM Query/Retrieve (Q/R). Although the 2 clinics included in this study used Varian ARIA as their R&V system, which does support DICOM Q/R (though it does not support automated export of Dose-Volume Histogram (DVH) data within the DICOM), we recognize that this is not representative of all clinical environments. One limitation of our proposed framework, therefore, is that many clinical systems do not support DICOM Q/R. To address this limitation, future work will focus on validating our automated data collection software, including the alternative data collection methods, at clinics with more diverse data sources and systems lacking DICOM Q/R capabilities. This effort aims to demonstrate the adaptability of our framework to environments where direct DICOM Q/R is not feasible. For such systems, data are exported through alternative methods (as shown in Fig. 2), such as using automated scripts to extract the data or manually exporting to an auxiliary PACS, which allows the CORE to link treatment records with planning data.

In addition, some legacy systems at these clinics archive data in proprietary formats, necessitating conversion to DICOM. This conversion is achieved either through custom code or by using an older version of the TPS software provided temporarily by the vendor, enabling us to open and export the data in DICOM format. Prior work on extracting radiation therapy data sets from proprietary archives likewise supports that conversion to DICOM-RT can be necessary to recover historic or otherwise inaccessible data, while also illustrating that such approaches entail nontrivial development and validation effort. By extending our data collection methods to these additional clinics, we aim to overcome one of the significant barriers in aggregating radiation therapy data at scale.

Despite its successes, this framework has important limitations. The current pipeline focuses on DICOM radiation therapy objects and does not automatically incorporate external clinical variables such as outcomes or toxicity, which must be obtained from separate clinical databases. Registry initiatives and minimum-data-element efforts emphasize that outcomes/toxicity and other cross-specialty elements (eg, disease status, staging, treatment intent) are integral components of learning health system applications, but they generally require connectivity beyond DICOM-RT alone. In addition, the system relies on the integrity of source records, such that errors or omissions in clinical documentation (eg, incomplete structure names or missing treatment details) may propagate into the aggregated database. These limitations motivate ongoing work toward improved standardization of data entry practices and future expansion of the framework to incorporate clinical outcomes and other non-DICOM data sources.

Practical implementation across diverse clinical environments also requires coordination with institutional information technology teams, particularly with respect to network configuration, firewall policies, secure DICOM communication, and provisioning of storage and compute resources for large-scale ingestion. Environments with limited support for DICOM query/retrieve necessitate auxiliary workflows that increase initial deployment complexity. Although these factors represent barriers to adoption, they are largely front-loaded during initial installation and can be mitigated through standardized deployment procedures and institutional support. Importantly, the ingestion framework is designed to operate in the background without disrupting routine clinical workflows, minimizing the burden on frontline clinical staff.

The present work addresses a foundational step in the development of data-driven radiation therapy applications: reliable and automated acquisition of comprehensive planning and delivery data sets. Further validation is required before downstream clinical applications can be broadly implemented. Future work should include multi-institutional evaluations of data completeness, consistency, and longitudinal stability. Prospective multicenter studies will be necessary to demonstrate the clinical utility and generalizability of tools developed on top of this infrastructure.

In its current form, the framework operates primarily as background infrastructure and does not directly alter the daily workflows of radiation oncologists, medical physicists, or therapists. However, as downstream applications are developed, different user groups may experience distinct benefits. Physicists and informaticians may benefit from improved access to standardized technical data sets for quality assurance and research, while clinicians may benefit indirectly from population-level insights and data-driven tools that support treatment planning and clinical decision-making. Consideration of these differing end-user perspectives will be important in guiding future interface design and clinical integration.

Our ongoing work with these clinics highlights the importance of developing flexible data extraction solutions that can accommodate a variety of clinical systems and data formats. It also underscores the need for broader adoption of standardized protocols like DICOM Q/R and for enhancements to the DICOM standard itself. Through this expanded effort, we hope to demonstrate that our framework can be effectively applied across diverse clinical settings, ultimately facilitating more comprehensive and scalable radiation therapy data aggregation.

## Conclusions

Multi-institutional data warehouses will take time to build. Although prospective data collection may be more straightforward, to reach a substantive number of cases, retrospective data must also be collected. Moreover, retrospective data present the greatest near-term opportunity to evaluate long-term clinical outcomes. Retrospective data ingestion is a complex, laborious task that necessitates automation and demands several layers of quality control and validation. Our software framework proved effective in unifying radiation therapy planning and delivery data from multiple clinical systems. Critical to this achievement was the ability to meticulously back-trace DICOM relationships, ensuring that only pertinent data were retrieved, thus optimizing data download efficiency. Furthermore, the framework exhibited exceptional resilience in the face of errors, system crashes, and network instabilities—a vital feature considering the prolonged periods required for data collection in each clinic. This robustness underscores the framework’s reliability and marks a significant step forward in the field of data consolidation in clinical environments.

## Declaration of interests

James Lamb reports receiving research funding from Siemens Healthineers; this funding is unrelated to the subject matter of this manuscript. Daniel Low reports receiving research funding from Siemens Healthineers, also unrelated to the subject of this manuscript. Additionally, Daniel Low serves on the Advisory Board of ViewRay; this role is likewise unrelated to the content of this manuscript.
